# Biomarkers for the Detection and Risk Stratification of Aggressive Prostate Cancer

**DOI:** 10.3390/cancers14246094

**Published:** 2022-12-11

**Authors:** Samaneh Eickelschulte, Anja Lisa Riediger, Arlou Kristina Angeles, Florian Janke, Stefan Duensing, Holger Sültmann, Magdalena Görtz

**Affiliations:** 1 Junior Clinical Cooperation Unit, Multiparametric Methods for Early Detection of Prostate Cancer, German Cancer Research Center (DKFZ), 69120 Heidelberg, Germany; 2 Department of Urology, University Hospital Heidelberg, 69120 Heidelberg, Germany; 3 Division of Cancer Genome Research, German Cancer Research Center (DKFZ), National Center for Tumor Diseases (NCT), 69120 Heidelberg, Germany; 4 Faculty of Biosciences, Heidelberg University, 69120 Heidelberg, Germany; 5 Molecular Urooncology, Department of Urology, University Hospital Heidelberg, 69120 Heidelberg, Germany; 6 German Cancer Consortium (DKTK), 69120 Heidelberg, Germany

**Keywords:** prostate cancer, early detection, risk stratification, tissue-based biomarkers, liquid-based biomarkers

## Abstract

**Simple Summary:**

Prostate cancer is a heterogeneous disease and a major cause of cancer deaths worldwide. The most widely used prostate cancer biomarker, prostate-specific antigen, lacks sensitivity and specificity in the diagnosis of malignant disease. Hence, novel tissue-based biomarkers have emerged for the detection and risk assessment of prostate cancer. Over the past years, liquid biopsy biomarkers introduced a new diagnostic concept to complement current tissue diagnosis strategies. Liquid biopsies non-invasively provide a characterization of heterogenous tumor profiles. Here, we highlight the most prominent tissue and liquid biopsy biomarkers for the detection and risk assessment of prostate cancer.

**Abstract:**

Current strategies for the clinical management of prostate cancer are inadequate for a precise risk stratification between indolent and aggressive tumors. Recently developed tissue-based molecular biomarkers have refined the risk assessment of the disease. The characterization of tissue biopsy components and subsequent identification of relevant tissue-based molecular alterations have the potential to improve the clinical decision making and patient outcomes. However, tissue biopsies are invasive and spatially restricted due to tumor heterogeneity. Therefore, there is an urgent need for complementary diagnostic and prognostic options. Liquid biopsy approaches are minimally invasive with potential utility for the early detection, risk stratification, and monitoring of tumors. In this review, we focus on tissue and liquid biopsy biomarkers for early diagnosis and risk stratification of prostate cancer, including modifications on the genomic, epigenomic, transcriptomic, and proteomic levels. High-risk molecular alterations combined with orthogonal clinical parameters can improve the identification of aggressive tumors and increase patient survival.

## 1. Introduction

Prostate cancer (PCa) is the second most frequent malignancy among men, accounting for 27% of all cancer diagnoses in males [1,2]. Although the survival rate of men with early diagnosed localized PCa can be as high as ~99%, men who are diagnosed with late-stage disease have a survival of only 30% over 5 years [3,4,5]. The detection of PCa is feasible using the blood-based biomarker prostate-specific antigen (PSA) and digital rectal examination (DRE) as a screening method. The diagnosis of PCa at an early stage has benefits, including the high possibility of cures, less aggressive treatment options, reduced disease progression to advanced or metastatic stages, and improved quality of life [6]. The widespread use of PSA as a screening method for PCa led to improved PCa diagnoses, with a shift towards earlier tumor stages. Within the European Randomized Study of Screening for Prostate Cancer (ERSPC), PSA screening was shown to reduce PCa death and metastasis in men aged 55–69 years by 29% and 30–42%, respectively [7,8].

However, this reduction in mortality and metastases is associated with considerable overdiagnosis and overtreatment rates of PCa. Moderately elevated PSA serum levels are controversial, as PSA is produced by the normal prostate, as well as PCa cells; therefore, they lack cancer sensitivity and specificity. Accordingly, PSA testing has led to many instances of men with elevated PSA levels with a subsequent negative PCa diagnosis. Considering the psychological burden of false-positive PSA values, only ~25% of men with elevated PSA (>4 ng/mL) have a positive prostate biopsy and show PCa incidences [9,10]. Non-invasive markers with the potential to distinguish between normal prostate and PCa could significantly reduce the number of invasive diagnostics in the form of prostate biopsies. A further challenge in PCa management is the lack of an adequate risk stratification scheme of detected PCa in order to guarantee an optimal, individually adapted therapy. Men with indolent tumor forms unlikely to progress to clinical significance require further diagnostic options [11]. The molecular characterization and analysis of tissue biomarkers are important for the risk stratification of PCa, complementing the histopathological evaluation of prostate biopsy samples [12,13]. Due to intra-tumoral heterogeneity, tumor molecular profiling by tissue biopsy is limited by finite sampling sites. Therefore, there is increasing interest in identifying tumor biomarkers in liquid biopsy samples to improve detection, clinical decision making, and clinical outcomes for patients. Being able to detect and evaluate the aggressiveness of PCa as early as possible can improve patient survival. This review aims to present a comprehensive overview on recent molecular markers for the early detection and risk stratification of aggressive PCa in both tissue and liquid biopsy.

## 2. Tissue Prostate Cancer Biomarkers

### 2.1. Genomic and Epigenomic Tissue Biomarkers

Whole genome sequencing (WGS) and characterization of genomic alterations revealed various novel tissue-based biomarkers associated with PCa [13]. On the other hand, epigenomic changes occur early during PCa development and are often acquired prior to somatic mutations or chromosomal aberrations [14]. Consequently, alterations in the epigenome have higher levels of occurrence compared to the most frequent genetic defects in PCa [15,16]. Both genomic and epigenomic alterations emerged as powerful tools to distinguish indolent tumors from those likely to progress.

#### 2.1.1. DNA Mutations and Copy Number Alterations 

**Figure 1:**
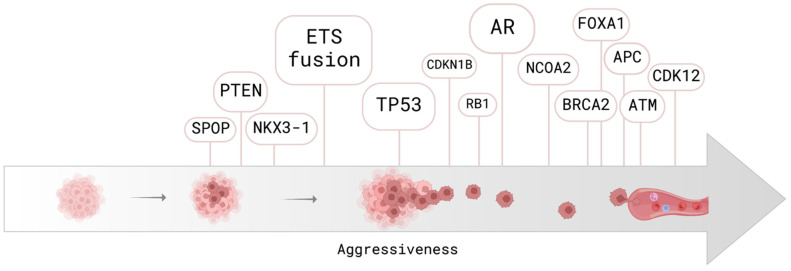
Common mutations detected in PCa are shown according to their occurrence along the disease progression. The bubble size indicates the frequency of mutation. Created with BioRender.com (Accessed on 18 October 2022).

Mutations in DNA damage repair (DDR) machinery are key mechanisms driving PCa tumorigenesis in a subset of prostate tumors [17,18]. Germline mutations in homologous recombination (HR)-mediated DDR genes, *BRCA1* or *BRCA2*, are relatively rare (<5%) in unselected patients, but important prognostic factors for PCa [19,20,21,22]. Mutations in DDR genes through HR have been shown to be associated with aggressive tumor growth and increase the risk of PCa at a younger age [20,23]. Interestingly, somatic mutations in genes involved in DDR (i.e., *BRCA1/2*, *ATM*, and *CDK12*) can be found at a higher frequency of ~20% [24,25] and, therefore, play a substantial role in the risk stratification of PCa. Mutations leading to a loss of function in the tumor suppressor genes, *TP53* and *RB1*, represent further genomic alterations associated with PCa progression [17,26,27,28,29]. *TP53* mutations are frequently observed in primary PCa [30] with high-risk features and have an important prognostic value in the early detection of PCa [31,32,33,34]. Mutations in the androgen receptor (AR) and alteration of AR-regulated signaling pathways are detected in about one-third of metastatic PCa patients, ranking as the most frequently reported genomic alterations in PCa [34,35,36,37]. Mutations in *SPOP, CHD1, PTEN, NKX3-1, FOXA1*, and *APC* are other known genomic alterations in primary and advanced PCa (Figure 1) [29,38,39,40,41,42].

The fusion of *TMPRSS2* and erythroblast transformation-specific (ETS)-related gene (ERG) is the most frequent (~50%) structural abnormality in PCa. Because of its high frequency and specificity, *TMPRSS2-ERG* is used as a diagnostic biomarker for PCa [43,44,45]. The *PTEN* loss often co-occurs with ERG rearrangements and is necessary for its carcinogenesis effect [20,46]. 

**Figure 2:**
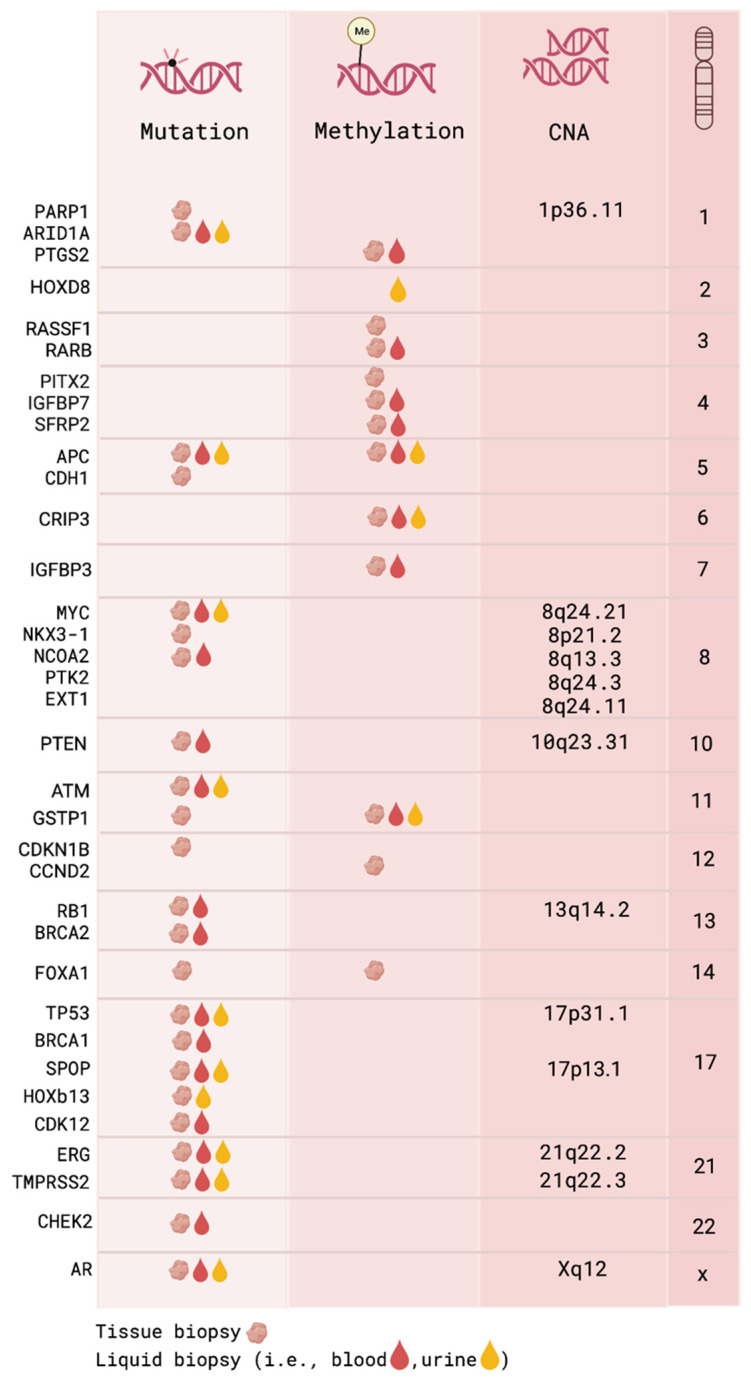
Common genomic and epigenomic instabilities detected in tissue, blood, and urine of PCa patients [17,29,35,39,55,56]. Created with BioRender.com.

Copy number alterations (CNAs), either alone or in combination with mutations, can be used as a potential biomarker for the risk stratification of aggressive PCa [17,47]. The CNA analysis may have clinical applications in the prognosis of PCa independent of the Gleason score. It has been shown that tumors carrying few CNAs had a more favorable prognosis than did those containing a high number of CNAs [36,48]. Chromosomal gains affecting *MYC, NCOA2*, and *AR* are known to be present in high-risk PCa [36,39,49]. The amplification of *MYC* has been reported as the most frequent copy number gain in primary PCa (up to 30% in advanced disease) [3]. Deletions in *GSTP1, CDKN1B*, and *ARID1A* have been shown to be associated with the risk of hereditary PCa and metastasis formation [50,51,52,53]. Figure 2 shows known driver mutations and CNAs detected in tissue that are involved in the initiation, as well as the progression, of PCa. Furthermore, mutations and CNAs in the mitochondrial genome (mtDNA), resulting in the dysregulation of mitochondrial homeostasis, can play a role in tumor initiation, growth, and metastasis. Mutations and CNAs in mitochondrially encoded *tRNA, CO1, ATP6, PC3,* and *ND* genes can be potential prognostic biomarkers that may improve the early detection of aggressive PCa [54]. Overall, the heterogeneity of PCa is mirrored by the presence of multiple genomic alterations along PCa’s development. Identifying mutations and CNAs is essential for using these valuable tools for the detection and risk assessment of PCa.

#### 2.1.2. DNA Methylation

DNA methylation, occurring at the 5’-carbon of cytosine residues (5-mC), is the most widely studied epigenomic modification in cancer. 5-mC resides at cytosine–guanine dinucleotides (CpGs), and the hypermethylation of promoter-associated CpG clusters, termed CpG islands, is linked to transcriptional repression [57,58]. Aberrant promoter hypermethylation and concomitant gene silencing have been observed at several genes in PCa, such as *GSTP1*, *RASSF1*, *APC, CCND2*, and *PITX2* (Figure 2). Their 5-mC levels, both as an individual biomarker or as a biomarker panel, were demonstrated to discriminate PCa from benign tissue with high sensitivity and specificity [59,60,61,62,63,64,65]. The combined analysis of *GSTP1*, *RASSF1*, and *APC* showed superiority relative to the histological assessment of biopsy specimens, detecting PCa in 62 and 68% of histologically negative biopsies in two independent, large-scale studies (498 and 350 biopsies analyzed, respectively) [60,62]. These findings highlight the applicability of 5-mC biomarkers to large patient cohorts and demonstrate their capability in detecting locally confined tumors. Abnormal DNA methylation is described as emerging progressively in PCa and may be involved in the formation of metastases [66,67]. Hence, various studies aimed to identify 5-mC biomarkers with prognostic significances that could identify aggressive tumors. *GSTP1*, *APC*, *RARB*, and *PITX2* are among the most extensively studied prognostic 5-mC biomarkers in Pca, and numerous reports emphasize their value as prognostic markers when analyzed in resected prostate tissues [61,64,65,68,69,70,71,72]. The promoter hypermethylation of those genes has been linked to the increased risk of clinical and biochemical recurrence (BCR) [68,69,70,71], as well as PCa-associated mortality [61,64,72]. Importantly, aberrant DNA hypermethylation events are also detectable in prostate tissue biopsies [63,73,74,75]. This permits risk stratifications prior to clinical interventions and may circumvent the overtreatment of indolent PCa. For instance, combined 5-mC intensities at *GSTP1*, *APC,* and *RASSF1* in 12-core prostate biopsy specimens increased significantly across Gleason and NCCN (National Comprehensive Cancer Network) risk categories, demonstrating an association between 5-mC signals and the established risk factors. Interestingly, this association was still evident when only cancer-negative biopsy cores were assessed [74]. Hence, 5-mC biomarkers might compensate biopsy sampling limitations and facilitate the identification of high-grade tumors in histologically occult biopsies. Further studies found significant associations between unfavorable outcome and *APC* hypermethylation, as well as increased *PITX2* methylation in high- versus low-risk prostate tumors in 6- and 12-core biopsies, respectively [63,73].

With technological advances and plummeting sequencing costs, genome-wide 5-mC profiling approaches gained popularity for the identification of prognostic biomarkers in PCa. In a population-based cohort of PCa patients following prostatectomy, methylation array analyses identified 42 CpG sites that stratified non-recurrent PCa from patients developing metastatic-lethal disease within 5 years. Eight of forty-two CpG biomarkers were subsequently validated in an independent cohort and improved the accuracy of predicting adverse outcome compared to the mere assessment of the Gleason score [76]. Another study used resected multifocal PCa and categorized the aggressiveness of individual foci based on 5-mC similarities relative to matched lymph node metastases. An aggressiveness classifier, integrating 5-mC signals at 25 CpG sites, separated aggressive (i.e., predicted origin of lymph metastasis) from indolent PCa foci. Classifier validation in the PCa samples of The Cancer Genome Atlas (TCGA) showed significant associations between the predicted aggressiveness and presence of lymph node metastases, as well as tumor stage [77]. 

These studies demonstrate that the integrative analysis of multiple 5-mC patterns exposes information about a patient´s tumor (e.g., cellular composition or 5-mC signatures of aggressiveness), which is missed by the evaluation of individual markers and may advance 5-mC-based PCa prognosis.

#### 2.1.3. Histone Modification

In eukaryotic cell nuclei, histones are abundant DNA-wrapped structures and the major protein components of chromatin. Histones play an essential role in the regulation of gene expression via a number of post-translational modifications (i.e., acetylation and methylation). The deregulation of tri-methylated histone H3 at lysine 27 (H3K27me3) is known to silence tumor suppressor genes *GAS2* and *ADRB2*, which are both involved in PCa progression [78]. Therefore, H3K27me3 alteration emerged as a new biomarker for aggressive PCa. H3K4me2 methylation contributes to the activation of a ubiquitin-conjugating enzyme complex, UBE2C, and can be used as a biomarker for PCa prognosis [79]. On the other hand, transcription factors, including SOX2, NR2F1, and NANOG, induce the hypomethylation and subsequently inactivation of H3 [80]. The acetylation, methylation, and ubiquitination of the histone-variant, H2A.Z, occur at active promoter sites and contribute to oncogene activation and, subsequently, the progression of PCa [81]. 

#### 2.1.4. RNA

The transcriptome represents a layer of the molecular landscape of cancer cells that is useful in the development of clinically relevant biomarkers. In a study that evaluated the landscape of fusion genes in 144 localized PCa, the abundance of the read-through fusion transcripts RP11-356O9.1-TTC6 and TBCEL-TECTA was associated with BCR. Tumors harboring both fusion transcripts had a significantly increased risk for relapse [82]. The transcriptome profiling of five PCa specimens discovered eight fusion transcripts enriched in tumors compared to normal prostatic tissue [83]. These read-through transcripts were as follows: TRMT11-GRIK2, SLC45A2-AMACR, MTOR-TP53BP1, LRRC59-FLJ60017, TMEM135-CCDC67, KDM4-AC011523.2, MAN2A1-FER, and CCNH-C5orf30. In a subsequent validation experiment on PCa with clinical outcome data after radical prostatectomy (RP), the increased expression of any of the eight fusion transcripts was associated with BCR. No fusion transcripts were detected in healthy tissue donors, and positive expressions of TRMT11-GRIK2, MTOR-TP53BP1, and LRRC59-FLJ60017 were observed exclusively in recurrent tumors.

Long non-coding RNAs (lncRNAs) are transcripts that are at least 200 nt in length, with minimal or completely without protein-coding potentials [84]. The earliest utilization of lncRNAs for clinical use in PCa began with the discovery of the prostate-specific PCA3 transcript, which promotes cell survival by activating the AR pathway [85]. Unlike PSA, PCA3’s expression remains unaffected in other prostate pathologies, such as chronic prostatitis and benign prostatic hyperplasia (BPH) [86]. The RNA-seq analysis revealed that the lncRNA SChLAP1 is differentially expressed between aggressive and indolent tumors, and significantly associated with clinical risk factors for aggressive diseases, including BCR and metastatic progression [87,88,89]. Other lncRNAs with prognostic potential in PCa are PCAT-1 and PCAT-14. PCAT-1 was highly expressed in high-grade localized tumors and in a subset of metastatic specimens [90]. The low expression of PCAT-14 distinguished malignant from benign tissues, as well as high- and low-grade local tumors [90,91].

MicroRNAs (miRNAs) are small (~22 nt) non-coding RNAs involved in eukaryotic post-transcriptional gene regulation [92]. In localized PCa, differentially expressed miRNAs in tissue have been evaluated as risk stratification biomarkers, based on BCR-free survival and overall survival as common ends [93]. In a systematic review of 215 publications, 120 unique miRNAs were identified as individual prognostic markers in localized PCa [93]. Seven miRNAs—let-7b-5p, miR128a-3p, miR-188-5p, miR-224-5p, miR-23a-3p, miR-23b-3p, and miR-34b/c—were significantly associated with disease progression. Aside from individual miRNAs, miRNA signatures have also been evaluated for prognostic utility in localized PCa. A metric based on the tissue abundance of miR-96-5p, miR-183-5p, miR-145-5p, and miR221-5p was able to distinguish aggressive tumors from non-aggressive Pca in a Swedish cohort [94]. A 3-miRNA prognostic classifier involving miR-185-5p, miR-221-3p, and miR-326 predicted BCR-free survival in independent cohorts [95]. A highly combined RNA score based on the tumor expression of the miR-17-92 cluster (miR-17-3p, miR-17-5p, miR-18a, miR-19a, miR-19b, and miR-92a) was associated with a shorter BCR interval [96]. A 4-miRNA ratio model based on miR-23a-3p, miR-10b-5p, miR-133a, and miR-374b-5p tissue expression was shown to be a significant predictor of BCR-free survival, independent of routine clinicopathologic variables, as well as PCa-specific survival [97]. 

Circular RNAs (circRNAs) are predominantly noncoding RNAs generated via alterative splicing events that form covalently closed loops [98,99]. A comprehensive characterization of the circRNA landscape in localized PCa has shown significant circRNA dysregulation in tumors [98]. The same study identified 171 circRNAs essential for PCa. Additionally, tumors found to harbor extreme deviations (i.e., abundance or depletion) in circRNA expression were associated with poor BCR-free survival. In a recent study, the biomarker utility of circRNAs in PCa was further investigated using multiple patient cohorts for validation [99]. Ultimately, five circRNAs—circZNF532, circCDYL2, circLPAR3, circELK4, and circMAN1A2—were verified as deregulated between high/low pT stages. Moreover, the expressions of circSLC45A4, circFAT3, and circSEMA3C were independently validated to be negatively correlated with the Gleason score. Finally, circMKLN1 repression was successfully validated to be associated with BCR.

### 2.2. Gene Expression and Proteomic Biomarkers 

Gene expression signatures can provide potential information for PCa prognosis. Importantly, AR acts as a transcription factor that regulates the expression of genes encoding cellular homeostasis and proteases that are important for prostate function. Proteases are excellent biomarkers in PCa as about half of the human proteases are expressed in the prostate gland [100]. *KLK3*, encoding PSA, is one of the members of the kallikrein-related peptidase family, and it is highly expressed in the prostate [101]. In addition to its widespread role in PCa diagnosis, *KLK3* is involved in the progression of tumors towards metastasis by supporting cell proliferation and angiogenesis [102]. In addition to *KLK3*, the high-level expressions of *CD133* and *GRHL2* were observed in PCa and increased during disease progression [103]. Studies suggested that the expression levels of another cell surface antigen, prostate-specific membrane antigen (PSMA), directly reflect the tumor stage and BCR [104]. The upregulation of *TMPRSS4* is known to be associated with PCa early invasion and metastasis. The expression of four other transcription factors, including SOX2, PROM1, SNAI2, and TWIST1, assists TMPRSS4 functions in early metastasis [105]. Using gene expression profiling tools by comparing normal and PCa samples, DMPK, KCNQ4, and WIF1 were shown to be downregulated [106]. In the same study, it was reported that the expression levels of *KCNQ4*, *WIF1*, *PLN*, and *F3* were associated with shorter survival. 

Data derived from targeted antibody-independent proteomic assays (PRISM-SRM assays) and mass spectrometry of PCa tissue proteome provide additional support to identify protein signatures for the risk stratification of PCa. In tissue, a few of the differentially expressed protein biomarkers are capable of predicting PCa progression. Comparing metastatic to non-metastatic PCa tissues, three differentially expressed proteins, including PARP, RDH11, and NDRG3, were shown to be involved in PCa progression [107]. A classifier was created using differentially expressed proteins in formalin-fixed parafilm-embedded tissue specimens. This classifier comprised a panel of five proteins (i.e., FOLH1, KLK3, TGFB1, SPARC, and CAMKK2) that are capable of stratifying PCa patients according to the risk of aggressiveness [108]. In a recent study, seven differentially expressed proteins (i.e., HSPA9 and HSPE1, NPM1, VCAN, SERBP1, MRPL3, and UQCRH) were successfully used as indicators of PCa progression and aggressiveness [109,110]. Using gene enrichment and immunohistochemical analyses, POSTN, CALR, and CTSD were found to be associated with PCa progression and short patient survival [111]. 

Furthermore, protein phosphorylation plays a critical role in cellular signaling pathways and is one of the multiple mechanisms that drives cancer progression. Alterations in the PCa phosphoproteome have been shown to shift metabolic programming and fuel cellular proliferation and metastasis [112]. AKT-mediated phosphorylation events involving leukemia inhibitory factor receptor (LIFR) and its downstream target, ERK2, activate a signaling cascade that promotes tumor growth and metastasis [113]. Therefore, the AKT/LIFR/ERK2 axis can be used as a potential biomarker for predicting the risk of PCa progression.

## 3. Biomarkers in the Tumor Microenvironment

The tumor microenvironment (TME) is a network consisting of supporting cellular and stromal components: (i) immune cells, including tumor-associated macrophages, and (ii) non-immune cells, such as endothelial cells, inflammatory cells, and cancer associated-fibroblasts (CAFs). Cancer cells remodel the stromal cells and transform the TME into a habitat capable of evading immune cell infiltration to enhance tumor development. The TME releases various factors into blood circulation, including chemokines, cytokines, and enzymes, that can be used as biomarkers for detecting PCa in early stages. 

Through accelerating the epithelial-to-mesenchymal transition (EMT) of cancer cells, stromal CAFs enhance the migration, proliferation, and invasiveness of PCa cells. Another regulator of the EMT pathway is transcription factor FOXA1. Mutation and post-translational modifications, such as methylation and acetylation, can alter FOXA1 activity and promote cancer progression (Figure 1) [114]. Studies have shown that miRNAs derived from extracellular vesicles (EVs) play an important role in tumor–stromal interactions [115]. miR-409 is located within the DLK1-DIO3 cluster and released from EVs. The upregulation of miR-409 was found to be associated with higher Gleason scores and PCa aggressiveness by targeting tumor suppressor genes (i.e., *Ras suppressor 1*, *tumor suppressor candidate 1,* and *stromal antigen 2*) [116]. Other DLK1-DIO3 cluster miRNAs (i.e., miR-154* and miR-379) have similar roles and actively take part in tumor-stroma interactions by inhibiting SMAD7 and stromal antigen 2, respectively [117]. SMAD7 is an antagonist of transforming growth factor-beta (TGF-β). TGF-β and growth and differentiation factor 15 (GDF15) play a crucial role in the production of collagen and the deposition of extracellular matrices (ECMs). Stromal cells are sources of TGF15, which can contribute to cancer progression driven by the TME [118,119]. 

Depending on the tumor stage and genetics, tumoral autophagy in the TME plays a divergent role (i.e., the suppression or promotion) in PCa development. mTORC is an important regulatory kinase involved in cell proliferation and autophagy. Genetic alterations, such as *PTEN* deletions, lead to the constitutive activation of mTORC, decrease autophagy, and encourage tumor growth and metastasis [120,121]. In addition, the expression levels of four major autophagy genes (*LC3A*, *LC3B*, *p62,* and *Beclin1*) are associated with higher Gleason scores and tumor aggressiveness [120,122]. The detection of these autophagy biomarkers may be of value for the risk stratification of PCa. 

AR and its ligands, testosterone and 5α-dihydrotestosterone, promote cellular signaling events that are essential for prostate growth and optimal function. Stromal cells express high levels of AR during prostate growth. In androgen ablation therapies, a low AR expression during the course of PCa progression is associated with BCR and disease progression [123]. In this scenario, PCa cells use other receptor transcription factors, such as the Glucocorticoid Receptor (GR), independently of AR. The upregulation of GR signaling promotes cell proliferation and differentiation and, therefore, has been associated with aggressive PCa [124]. 

## 4. Liquid Biopsy Biomarkers in Prostate Cancer

### 4.1. Cell-Free DNA 

Liquid biopsies emerged as a powerful tool for the early identification of aggressive PCa. Cell-free DNA (cfDNA) isolated from bodily fluids (e.g., blood, urine, saliva, or cerebrospinal fluid) is well known to contain tumor-derived molecular information and has been used for cancer diagnosis, genotyping, prognosis, and disease monitoring in various tumor entities [125,126,127,128]. In contrast to tissue samples, liquid biopsies represent less procedural risk to the patient and are not spatially restricted. A cfDNA sample may contain tumor DNA from multiple (if not all) cancerous lesions and could overcome sampling limitations posed by conventional tissue biopsies [129,130]. Hence, biomarkers for aggressive Pca could be obtained repeatedly and with a lower risk of sampling bias and undersampling. Data derived from a study using 45 plasma samples from Pca patients showed that the circulating tumor DNA (ctDNA)-based analysis in liquid biopsy is capable of identifying all driver DNA modifications present in matched solid biopsy [56]. Promising results were obtained in advanced or late-stage Pca, but the detection of early stage Pca using liquid biopsies is still limited due to the low quantity of ctDNA [131,132].

#### 4.1.1. Total cfDNA Amount and Integrity

The analysis of the total cfDNA content can serve as a first diagnostic tool prior to the analysis of more specific genomic (i.e., mutations and CNAs) and epigenomic (i.e., methylated DNA and histone modifications) alterations. The total amount of detectable cfDNA and, particularly, ctDNA arising from tumor cells can vary widely between different tumor entities. Several studies indicate that the higher plasma cfDNA concentrations in Pca patients are correlated to a positive biopsy, high Gleason score, PSA recurrence, and shorter BCR-free survival [133,134]. 

In addition to cfDNA quantification, a more detailed characterization of the cfDNA fragments can be applied to further improve the sensitivity of tumor detection. In plasma, cfDNA fragment profiles harbor a characteristic peak at 167 bp. It has been shown that cfDNA fragment lengths are slightly shorter in cancer patients, outlining the potential of fragmentation analyses for tumor diagnostics [135,136,137,138]. CfDNA integrity, determined by the quantification of the *ALU* gene (i.e., *ALU* 247 bp ratio to *ALU* 115 bp), is high in PCa patients and can distinguish PCa patients from BPH patients with elevated PSA [139,140]. Additionally, the ratio of the *PTGS2* DNA fragment (small fragment size < 200 bp, associated with apoptosis) to the *Reprimo* DNA fragment (larger fragment size > 250 bp, associated with other types of cell death) was used to define an apoptosis index [141]. This index distinguishes between PCa patients with localized disease and BPH patients and shows a significant correlation to BCR. Interestingly, seminal fluids from PCa patients contain significantly longer cfDNA fragments (>1000bp) compared to BPH patients and healthy controls [142]. 

#### 4.1.2. Genomic Analysis of cfDNA

The WGS analysis of cfDNA revealed multiple mutations and CNAs consistent with PCa tissue analyses: *BRCA1/2*, *TP53*, *SPOP*, *ATM*, *APC*, and *CHEK2* mutations, as well as CNAs in *TMPRSS2-ERG* and *RB1,* and *PTEN* deletions, *AR* and *MYC* amplification (Figure 2) [55,56,143,144,145,146,147,148,149,150]. *AR* and its constitutively active splice variants, *AR-V7* and *AR-V3*, are associated with poor survival and are used as potential biomarkers in PCa [151,152]. In addition to *AR*, *TP53* mutations and *TMPRSS2-ERG* fusion are among the most frequently altered genes in PCa (Figure 1). Since *BRCA1/2* are involved in DDR machinery, existing mutations in *BRCA1/2* increase further somatic mutation rates, as well as CNAs, and they are considered high-risk biomarkers for PCa progression [153].

The loss of heterozygosity (LOH) in specific chromosomal regions detected by short tandem repeats (STR; microsatellite DNA) can be used to distinguish PCa and BPH patients at the time of the initial diagnosis [154,155]. D8S360 is a STR marker that discriminates between metastatic and localized disease, as metastatic PCa carries a higher frequency of LOH in D8S360 [154,156]. It has been reported that the LOH frequency at 14 microsatellite markers was linked to risk factors, such as tumor stage, Gleason score, and metastatic disease [156]. 

Applying tagged-amplicon deep sequencing on the plasma samples of patients with localized PCa, *TP53* variants were identified in 22 of 189 cases (12%), and 14 out of 21 detectable *TP53* variants were predicted to be pathogenic or likely pathogenic [157]. Patients with the detectable *TP53* ctDNA variant harbored a significantly shorter metastasis-free survival [157,158]. Both studies highlight the potential of ctDNA detection for early diagnosis or risk stratification in PCa.

#### 4.1.3. cfDNA Methylation 

Various reports demonstrated that hypermethylation events at genes with prognostic implications in PCa tissue (e.g., *GSTP1*, *APC*, and *PTGS2*) are also detectable in plasma, serum, or urine samples [159,160,161,162,163,164,165,166]. Different liquid biopsy-based 5-mC biomarker panels showed positive correlations between 5-mC levels and the increasing Gleason score [159,165,166], suggesting the possibility of minimal invasive risk stratification. For instance, a urinary 4-gene 5-mC panel (i.e., *GSTP1*, *APC*, *CRIP3*, and *HOXD8*) was found to predict disease progression in patients with Gleason 6 PCa under active surveillance [166]. These results illustrate that aberrant 5-mC signatures in urine may be of value for identifying men that could benefit from initiating active therapy. Another study associated 5-mC levels at six genes (i.e., *GSTP1*, *SFRP2*, *IGFBP3*, *IGFBP7*, *APC*, and *PTGS2*) with increased Gleason scores, as well as clinical risk categories, and demonstrated that urine 5-mC markers can detect high-grade lesions missed by tissue biopsies (Figure 2) [165]. Despite these promising results, the detectability of tumor-derived 5-mC signals in liquid biopsies remains limited, especially in localized PCa [160,161]. This might be overcome by expanding the number of assessed biomarkers using genome-wide approaches. However, only a few studies have employed genome-scale methylome profiling relative to cfDNA samples so far due to the low DNA quantities present in most plasma and urine samples [167,168,169]. Although these studies focus mostly on advanced PCa, their results are encouraging for applications in localized disease. As an example, Moss and colleagues showed that prostate-specific 5-mC signals can be detected in plasma cfDNA via a tissue-of-origin deconvolution approach and linked the prostate-specific 5-mC levels to therapy resistance/sensitivity [168]. This suggests that cellular compositions with potential prognostic values can be inferred from PCa cfDNA samples [170]. Currently, whole-methylome-profiling techniques are adapted to low DNA input quantities (e.g., cfMeDIP-seq [171,172], cfMBD-seq [173,174], cfTAPS [175], and cfMethyl-seq [176]), allowing their usage on cfDNA samples. For example, a recent finding revealed hypermethylation in metastasis, compared to localized PCa using cfMeDIP-seq technology, and provided valuable insights into the 5-mC-dependent mechanisms of tumor aggressiveness [177]. 

### 4.2. Cell-Free RNA

An analysis of cell-free RNA (cfRNA) molecules enables the minimally invasive detection of PCa in biofluids including blood, urine, and seminal fluids. Urine-based biomarker tests using RNA analytes are similarly being applied for risk estimation. The ExoDX Prostate IntelliScore (Exosome Diagnostics) assesses tumor grades based on the abundance of the PCA3, ERG, and SPDEF RNA from a first catch, non-DRE urine specimen [178].

In a validation cohort of 519 patients, the 3-gene (i.e., *SPDEF, ERG,* and *PCA3*) assay discriminated high-grade tumors (Gleason score ≥ 7) from low-grade and benign tumors [179]. The Mi-Prostate Score integrates TMPRSS2-ERG and PCA3 RNA levels in DRE urine samples with serum PSA abundance. The incorporation of urine TMPRSS2-ERG and PCA3 abundance, independently or in combination, outperformed the risk prediction model for localized diseases based on serum PSA alone [180]. Another disease risk score, utilizing combined HOXC6 and DLX1 mRNA levels in post-DRE urine and traditional clinical risk factors, was successful in detecting high-grade and clinically significant (Gleason score ≥ 7) PCa [181]. 

The combined circulating transcript levels of GOLM1, NKX3-1, and TRPM8 were shown to be able to stratify low- and high-risk PCa [182]. Aggressive tumors were defined to present extracapsular extension with advanced tumor stage (≥pT3). The 3-mRNA markers outperformed both the Gleason score and PSA in identifying high-risk PCa. 

In a study involving a cohort of 100 PCa patients and 50 men with BPH, 14 miRNAs were consistently abundant in the serum samples from patients with low-grade PCa or BPH, while high-grade PCa had significantly reduced levels [183]. A score based on the serum abundance of all 14 miRNAs was predictive of the absence of high-grade PCa in the cohort. Moreover, combining the miRNA signature with BCR data resulted in the accurate classification of low-risk PCa. A 3-miRNA signature (miR-222-3p, miR-24-3p, and miR-30c-5p) detected in urine was identified in cohort 1, and it was validated in cohort 2 that it could distinguish BPH samples from PCa specimens. An additional 3-miRNA signature (miR-125b-5p, let-7a-5p, and miR-151-5p) was simultaneously identified in cohort 2—and validated in cohort 1—which can predict time to BCR independent of clinicopathologic features after RP [184]. The plasma levels of miR-20a and miR-21 were found to be associated with a high Cancer of the Prostate Risk Assessment score [185]. Moreover, the combination of four miRNAs (i.e., miR-20a, miR-21, miR-145, and miR-221) was able to classify low-and high-risk disease, which were initially assessed using the D’Amico risk classification score [186]. In another study, a high-risk disease was identified based on the Gleason score, pathological T stage, surgical margin status, and diagnostic PSA. The plasma levels of miR-17-5p and miR-106a-5p, as well as miR-20a-5p and miR-20b-5p, were shown to be associated with high-risk diseases [187]. These results were also consistent with the miRNA expression and BCR-free survival data from the TCGA dataset. 

### 4.3. Extracellular Vesicles

Nucleic acids and proteins isolated from EVs are potentially useful biomarkers for the diagnosis, detection of tumor origin, and risk stratification of PCa [188]. Four PCa-related miRNA (miR-141, miR-145, miR-221, and miR-451a) and the known PCa mRNA biomarkers, PCA3 and TMPRSS2-ERG, could be quantified in plasma or urinary exosomes from PCa patients [189,190]. Further studies identified GATA-binding protein 2 (GATA2) transcripts in urinary exosomes as additional biomarkers for the detection and risk stratification of PCa. GATA2 mRNA is secreted by PCa cells and known to play a role in PCa development and aggressiveness. The combination of all three biomarkers further improved PCa detection compared to a single biomarker evaluation [191]. 

In a comprehensive proteomic analysis of EVs from urine and PCa tissue, the majority of proteins identified in EVs originating from PCa tissue were also detectable in urinary EVs (94.69%), indicating that the proteomic analysis of urinary EVs reflects the proteome of EVs in PCa tissues. The comparison of exosomal content in urine samples from PCa patients before and after the initial treatment showed that prostate-specific proteins (e.g. KLK2, KLK3/PSA, FOLH1, MSMB, ACPP, TGM4, NDRG1, and NKX3-1) and androgen-regulated genes (e.g., *FKBP5*, *FAM129A*, *RAB27A*, *FASN*, and *NEFH*) were significantly reduced in post-treatment urine samples, which were in turn significantly enriched in EVs containing bladder- and kidney-associated proteins [192]. In a proteomic analysis of urinary exosomes, eleven proteins (SCIN, AMBP, FABP5, CHMP4C, CHMP2B, BAIAP2, GRN, SYTL2, CALR, CHMP4A, and DNPH1) showed significant enrichments in the urine samples of PCa patients versus patients with negative biopsy [193]. Fatty-acid-binding protein 5 showed significant higher levels in PCa samples compared to patients with negative biopsy, and it was significantly associated with the Gleason score for the prediction of PCa. 

### 4.4. Circulating Tumor Cells and Secretome

Circulating tumor cells (CTCs) are secreted from primary and/or metastatic tumor sites; they enter the circulation and are responsible for tumor metastasis. The presence of CTCs in the peripheral blood of cancer patients is associated with decreased patient survival [194]. Due to low numbers in non-metastatic tumors, CTCs are detected and counted using enrichment methods [195]. Carrying the majority of mutations present in PCa tissues, CTCs can provide useful prognostic information for PCa detection at early stages. 

The single cell isolation and subsequently amplification of the whole genome, immunostaining, next generation sequencing (NGS), and fluorescence in situ hybridization analyses have been used to characterize genomic aberrations of CTCs. The assessment of a DNA-based signature in CTCs revealed genomic alterations, such as a loss in tumor suppressor genes *TP53*, *PTEN*, *NKX3-1*, *CDKN1B,* and *RB1*. Losses in *TP53*, *PTEN,* and *RB1* were linked to PCa aggressiveness [28,196]. Common chromosomal gains in PCa include the amplification of genes coding the focal adhesion kinase *PTK2*, *NCOA2,* and *MYC* [196,197]. CNAs in *NCOA2* offer prognostic values for the identification of aggressive PCa [49]. Additionally, *AR* and *EXT1* are the other affected genes with gains in the chromosomal regions. CTCs in aggressive PCa possess large-scale transition scores, as well as DNA-SCARS as genomic alterations [196].

The proteomic analysis of CTCs showed a number of inflammatory cytokines, proteases, and glycoproteins that can potentially be used as biomarkers. An elevated level of IL-8 has been observed in cell lines with invasive behavior, compared to those less likely to progress [198]. Matrix metallopeptidases (MMPs) are a group of zinc-dependent endopeptidases known as facilitators of tumor invasion and metastasis via degrading connective tissue barriers [55,199]. Analyzing CTC-secreted MMPs revealed higher levels of MMP-2 and MMP-9 expression in aggressive compared to indolent PCa [199,200]. A quantitative mass spectrometry-based approach showed that the urinary glycoprotein, prostatic acid phosphatase (ACPP), has discrimination power for distinguishing aggressive PCa from non-aggressive PCa [201]. The urinary levels of 8-OHdG and 8-iso-PGF2α involved in oxidative DNA damage are also shown to be associated with PCa’s progression [202]. Similarly to tissues, the elevated PSMA expression in CTCs was correlated with tumor grade and PCa progression [104,203]. This is equally the case for CD133, as its overexpression was indicative of progressive late-stage PCa [204]. A systematic review of 43 publications, representing CTCs analyses impact on non-metastatic PCa, reported the utility of CTCs as potential prognostic markers in localized PCa. Nonetheless, validation studies are required to stablish the sensitivity and specificity of CTCs for the characterization of early stage PCa [205]. 

### 4.5. Tumor-Educated Platelets

Tumor-Educated Platelets (TEPs) reprogram the behavior of CTCs via a direct interaction accompanied by exchanging RNA, lipids, and proteins (i.e., beta-3 integrin marker CD61) [206]. This trafficking event creates a supportive microenvironment for CTCs and facilitating their immune evasion via protecting them from natural killer cells [207]. The ability of platelets to alter tumor cells’ phenotypes and genetic content promote cell growth and plasticity [206]. During the course of tumor metastases initiation, TEPs facilitate EMT to lead the tumor towards metastases [206]. Hence, TEPs biomarkers can present adjuvant information for the early diagnosis of PCa. For instance, the detections of TEP biomarker transcripts (i.e., KLK2, KLK3, and FOLH1) were shown to be associated with high-level serum PSA and poor survival [208]. 

## 5. Importance of Bioinformatic Tools in Prostate Cancer Detection and Risk Stratification

The development of high-throughput technologies, such as NGS, markedly increased our knowledge towards exploring cancer biomarkers, and subsequently facilitated the detection and risk assessment of tumors. Despite having a promising future in development of precision medicine, these technologies generate a massive volume of multiple data streams. Bioinformatic pipelines offer computationally efficient and reliable prospects for analyzing and studying the sequencing data, allowing the development of a sensitive and specific system for PCa management operations. 

In addition, PCa intra-tumoral heterogeneity and its following challenges in early tumor detections can be relatively overcome by bioinformatic-assisted workflows. For instance, projects such as TRACERx focus on cancer evolutionary dynamics (i.e., the relationship between prostate tumor heterogeneity and tumor stage) via the characterization of chromosome instability [209,210]. DriverSub, PyClone, and SciClone are examples of novel bioinformatic approaches for the identification of tumor-derived mutations [210,211]. Furthermore, the multiple gene expression profiles of public datasets via the application of bioinformatic analysis identified seven genes (i.e., *BCO1, BAIAP2L2, C7, AP000844.2, ASB9, MKI67P1,* and *TMEM272*) involved in the PCa’s early prognosis and risk stratification [212]. 

## 6. Multi-Parametric Methods for Identifying Aggressive Prostate Cancer

**Figure 3:**
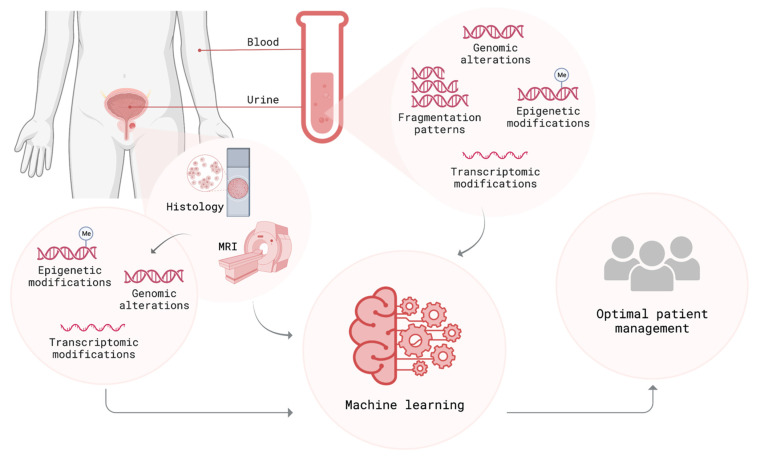
Multi-parametric approach converges the data derived from several sources (i.e., tissue and liquid biopsy, as well as MRI and histology) and uses them as an input for machine learning algorithms. Subsequent to this validation, this approach offers great potential to create a comprehensive patient profile and improves clinical outcomes. Created with BioRender.com (Accessed on 9 November 2022).

A major challenge in the early diagnosis of PCa using liquid biopsies is the low-abundance of tumor-derived genetic signatures (i.e., cfDNA and cfRNA). Overcoming this requires high-sequencing coverages to retrieve cancer-informative signals [136]. In addition, only a minor fraction of ctDNA exhibits genomic alterations, particularly at the early stage of cancer development. Therefore, studies sought to integrate different diagnostic/prognostic techniques to establish cancer-informative parameters and to improve the detection of tumor-specific signals. In this regard, there are two possibilities to interpret the data derived from PCa examinations: (i) combining different diagnostic and prognostic approaches (i.e., tissue biopsy, blood, and urine from liquid biopsy, imaging, and histopathology), and (ii) integrating different liquid biopsy profiles (i.e., genomic, transcriptomic, and epigenomic). In support of this, artificial intelligence (AI) can enhance the diagnostic expertise of clinicians for PCa risk stratifications (Figure 3). Further prioritization of biomarker candidates using established data repositories, such as the Cancer Genome Census (CGC), could also be performed before undertaking further validation studies [213]. 

### 6.1. Integration of Different Diagnostic/Prognostic Approaches

Although data integration from different diagnostic and prognostic approaches is relatively new, a few attempts have been made to combine genomic signatures and imaging techniques to improve PCa risk assessments. The combination of genomic analysis (i.e., CNAs), multiparametric MRI (mpMRI), and ^68^Ga-PSMA-PET/CT imaging resulted in a strong correlation between imaging features and genomic index lesions [214]. Using public genomic data, bioinformatic tools can compare mpMRI-visible and mpMRI-invisible lesions at a genomic level to underpin the genomic basis of PCa [215].

On the other hand, liquid biopsy data and prostate imaging enable the integration of multiple data streams into powerful multidisciplinary applications that can improve PCa detection. Recently, this approach advanced beyond pilot studies with a new study integrating CT-based radiomic analysis and liquid biopsy signatures from patients with advanced PCa. Accordingly, CTC counts, plasma cfDNA levels, and specific genomic alterations have shown strong correlations with the radiomic analysis of CT scans [216]. In addition, the implementation of AI and sequencing data derived from both tissue and liquid biopsy resulted in the creation of a sensitive and specific system that non-invasively facilitates PCa management (Figure 3) [217,218]. This system can add prognostic value for the differentiation of indolent and aggressive PCa.

### 6.2. Multi-Parametric Approach within Liquid Biopsy Profiles 

Information derived from multiple liquid biopsy profiles (i.e., genomic, epigenomic, transcriptomic, and fragmentomic) has the potential to introduce additional prominent biomarkers for the detection and risk assessment of PCa. A new study applied genomic, epigenomic, and fragmentation metrics in liquid biopsies to advance the use of liquid biopsy in pediatric cancer [219]. In addition, such multi-omics approaches can facilitate the characterization of heterogenous tumors [220]. For instance, cell-type compositions within tumors are estimated by an in silico method, epigenomic deconvolution (EDec), that combines DNA methylation and gene transcription profiles [221].

Other studies either combined the characterization of CTCs (enumeration and/or RNA analysis) with other analytes (i.e., cfDNA and ctDNA) [222,223,224,225] or performed the profiling of cfDNA and cfRNA to assess genomic alterations, along with transcriptomic aberrations [226]. For instance, an integrative multianalyte approach combined urinary methylation markers from men with a clinical suspicion for PCa with cfRNA quantification from EVs and clinical parameters. Applying a machine learning algorithm, this multivariable prediction model was capable of predicting the presence of PCa [227]. A multimodal genomic and epigenomic approach determined the molecular profile of low-abundance ctDNA via combining somatic mutation, fragmentation, and methylation analyses on cfDNA of patients with head and neck squamous cell carcinomas. Interrogating multiple methods confirmed an association between detectable ctDNA and the risk of having advanced diseases, as well as worse overall survival [228]. As a next step, future studies can employ independent multiparametric approaches to create a comprehensive model that has the potential to non-invasively improve early diagnosis, risk assessment, treatment monitoring, and clinical outcomes for aggressive tumors.

## 7. Conclusions

Despite its limitations, tissue biopsy remains the preferred tool for the detection and risk assessment of prostate tumors. The multitude of liquid biopsy biomarkers can play a complementary role for the non-invasive detection of PCa and can provide more insights into the differentiation of indolent and aggressive tumors. The identification of high-risk genomic, epigenomic, transcriptomic, and proteomic alterations, combined with orthogonal clinical parameters, can potentially improve the diagnosis of aggressive tumors and can increase patient survival. Integrating tissue, liquid biopsy, and other diagnostic/prognostic modalities, such as imaging, along with the clinical pathological profiles and novel machine learning approaches, could provide the necessary information for optimal patient management.
